# Efficiency and Safety of CyberKnife Robotic Radiosurgery in the Multimodal Management of Patients with Acromegaly

**DOI:** 10.3390/cancers15051438

**Published:** 2023-02-24

**Authors:** Carlos Alfonso Romero-Gameros, Baldomero González-Virla, Guadalupe Vargas-Ortega, Ernesto Sosa-Eroza, Mario Enrique Rendón-Macías, Lourdes Josefina Balcázar-Hernández, Moises Mercado, Novelthys Velasco-Cortes, Carlos Aaron Rodea-Ávila, Luis Flores-Robles, José Armando Lorenzana-Hernández, José Vázquez-Rojas, Margarita López-Palma

**Affiliations:** 1 Otorhinolaryngology Service, Hospital de Especialidades, Centro Médico Nacional Siglo XXI, Instituto Mexicano del Seguro Social, Mexico City 06720, Mexico; 2 Endocrinology Service, Hospital de Especialidades, Centro Médico Nacional Siglo XXI, Instituto Mexicano del Seguro Social, Mexico City 06720, Mexico; 3 Department of Biostatistics, Faculty of Health Sciences, Universidad Panamericana, Mexico City 03920, Mexico; 4 Medical Research Unit in Endocrine Diseases, Hospital de Especialidades, Centro Médico Nacional SigloXXI, Instituto Mexicano del Seguro Social, Mexico City 06720, Mexico; 5 Robotic Radiosurgery Service, Hospital de Oncología, Centro Médico Nacional Siglo XXI, Instituto Mexicano del Seguro Social, Mexico City 06720, Mexico

**Keywords:** acromegaly, radiosurgery, radiotherapy, pituitary neoplasms, somatomedins

## Abstract

**Simple Summary:**

Radiosurgery as an adjuvant treatment for acromegaly has shown efficacy in endocrine and tumor biochemical control, with an acceptable safety profile; however, the reported endocrine and tumor control rates and safety profile are heterogeneous. Therefore, the aim of the study was to evaluate the results of the efficiency and safety of radiosurgery in a well-characterized cohort of acromegalic patients, in addition to analyzing the prognostic factors associated with disease remission. We found a statistically significant reduction in IGF-1, IFG-1 x ULN, and GH concentrations at one year, and at the end of follow-up; in addition, it was observed that high basal IGF-1 concentrations were predictors of the biochemical absence of remission. We did not observe cases of optic neuritis associated with radiation toxicity or stroke.

**Abstract:**

Objective: To analyze, in a cohort of acromegalic patients, the results of the efficiency and safety of radiosurgery (CyberKnife), as well as the prognostic factors associated with disease remission. Material and methods: Observational, retrospective, longitudinal, and analytical study that included acromegalic patients with persistent biochemical activity after initial medical–surgical treatment, who received treatment with CyberKnife radiosurgery. GH and IGF-1 levels at baseline after one year and at the end of follow-up were evaluated. Results: 57 patients were included, with a median follow-up of four years (IQR, 2–7.2 years). The biochemical remission rate was 45.6%, 33.33% achieved biochemical control, and 12.28% attained biochemical cure at the end of follow-up. A progressive and statistically significant decrease was observed in the comparison of the concentrations of IGF-1, IFG-1 x ULN, and baseline GH at one year and at the end of follow-up. Both cavernous sinus invasion and elevated baseline IGF-1 x ULN concentrations were associated with an increased risk of biochemical non-remission. Conclusion: Radiosurgery (CyberKnife) is a safe and effective technique in the adjuvant treatment of GH-producing tumors. Elevated levels of IGF x ULN before radiosurgery and invasion of the cavernous sinus by the tumor could be predictors of biochemical non-remission of acromegaly.

## 1. Introduction

Pituitary tumors account for 20% of all intracranial tumors [1]. Functioning adenomas account for more than 50% of pituitary tumors [2] and are associated with clinical syndromes with significant morbidity and mortality and mechanical compression effects on vital structures [3]. Acromegaly is a chronic, deforming disease resulting from an excess production of growth hormone (GH), in most cases caused by a pituitary macroadenoma, which is presented clinically with a generalized acro-growth of soft tissue and bone. Untreated acromegaly has been associated with a greater number of metabolic, cardiovascular, osteoarticular, pulmonary, and neoplastic comorbidities. Patients with active acromegaly have excess mortality compared to the general population, which is associated with neoplastic and cardiovascular causes, and ultimately a progressive reduction in life quality [4,5,6].

Due to the heterogeneity and complexity in the presentation of the clinical picture, and low biochemical control/cure rates with the different medical–surgical strategies, most patients benefit from a multimodal treatment [7]. The initial treatment of acromegaly is surgical, through a transsphenoidal or transcranial approach, aimed in most cases at tumor demassification. In the absence of biochemical control after surgery and structural tumor remnant, adjuvant medical treatment with first- (Octreotide LAR and Lanreotide autogel) and second-generation (Pasireotide) somatostatin analogues is indicated [8]; the observed biochemical control rates of this treatment vary in the different published studies, ranging from 35–76% for the first-generation analogues, and from 26.9% to 93.3% for Pasireotide [9]. The biochemical control rates reported with dompaminergic agonists and Pegvisomant are of 50% [10] and 58–97% [9], respectively; however, the use of Pegvisomant implies high costs for health systems, something that could be unsustainable in developing countries.

The use of second- and third-line treatments, such as fractionated stereotactic radiotherapy and radiosurgery, have shown efficacy in endocrine and tumor biochemical control, with an acceptable safety profile. However, the rates of endocrine and tumor control and safety profile in their different modalities (LINAC, CyberKnife and GammaKnife) are heterogeneous [11]. Therefore, the aim of the study was to analyze, in a well-characterized cohort of acromegalics, the efficacy and safety of radiosurgery, as well as to see which prognostic factors were associated with disease remission.

## 2. Materials and Methods

An observational, retrospective, longitudinal, and analytical study was conducted in which acromegaly patients from the clinic of the Hospital de Especialidades, Centro Médico Nacional Siglo XXI, in Mexico City, Mexico, who received medical care during the period between 2010–2020, were included. The present study was approved by the local ethics and research committee (Registry identifier: R-2018-3601-149) and was consistent with the ethical guidelines of the 1975 Helsinki Declaration and the Mexican General Health Law on Research for Health Studies.

The acromegaly clinic was established in 2000 and currently has more than 600 patients who receive a uniform follow-up, according to established protocols with neuroendocrinological, neuro-ophthalmological and neurosurgical care. All patients, except known diabetics, underwent an oral glucose tolerance test, during which both GH and glucose were measured at baseline and at 30, 60, 90, and 120 min after intake of a 75 g glucose load. Additionally, according to our standardized protocol, insulin-like growth factor 1 (IGF-1), as well as morning cortisol, thyroid-stimulating hormone (TSH), free T4, prolactin (PRL), luteinizing hormone (LH), follicle stimulating hormone (FSH), and testosterone or estradiol were measured in the initial blood sample [12]. Patients received multimodal treatment including transsphenoidal or transcranial surgery, medical treatment with first generation somatostatin analogues (SSA) (Lanreotide autogel 120 mg deep subcutaneous application every 28 days and Octreotide LAR 20 mg intramuscular application every 28 days) and dopaminergic agonists (DA) (Cabergoline 1.5 to 3 mg orally weekly), fractionated stereotactic radiotherapy, and/or radiosurgery. The sample was obtained by non-probabilistic sampling of consecutive cases.

The collection of sociodemographic data, medical history, and laboratory and imaging data was carried out through a review of the patients’ electronic records. The inclusion criteria were patients with biochemical activity of acromegaly, of either sex, older than 17 years, and if they were candidates for radiosurgery treatment according to the current guidelines at the time of patient assessment [13,14]. The selection criteria for referral to radiosurgery were persistent biochemical activity (GH > 1 ng/mL and/or an IGF x ULN > 1.2) after surgical and medical treatment, tumor remnant < 30 mm, and distance from the tumor remnant to the optic chiasm > 3 mm [14,15]. The exclusion criteria were patients without complete data in their electronic files regarding biochemical and imaging outcomes related to the disease and patients who received other types of pituitary/cranial radiation therapy.

The primary outcome to be assessed was a biochemical remission of acromegaly at the end of the follow-up as a dichotomous nominal variable. Biochemical activity was defined as the presence of GH > 1 ng/mL and/or IGF x ULN >1.2, biochemical remission after radiosurgery as GH ≤ 1 and IGF x ULN 1.2 without medical treatment, and post-surgery biochemical control that required medical treatment with first-generation somatostatin analogues and that achieved the control goals. The IGF-1 X Upper Limit Normal (IGF-1 X ULN) value was obtained through the quotient of the IGF-1 obtained from the patient at the time of evaluation and the IGF-1 standardized for age and gender [16]. Tumor volume was calculated using the Di Chiro–Nelson method [17]. The invasion of the cavernous sinus was evaluated according to the Knosp classification. Local tumor control (LC) was defined as the containment and/or non-growth of the tumor remnant. Baseline variables were considered as those measured immediately before radiosurgery. Delay in radiosurgery time was defined as the time between the last surgery and the application of radiosurgery. Delay in diagnosis was defined as the time between the appearance of the first symptoms and the biochemical and imaging diagnosis of acromegaly. Panhypopituitarism was defined as the presence of three or more affected hypothalamic–pituitary axes. Diabetes and prediabetes were defined according to the American Diabetes Association criteria [18]. Hypertension was considered if the systolic blood pressure reading exceeded 140 mmHg or the diastolic was above 90 mmHg. Hypercholesterolemia and hypertriglyceridemia were defined as when values exceeded 200 and 150 mg/dL, respectively. Central hypocortisolism was defined by a cortisol concentration < 5 μg/dL at 7:00 h. Central hypothyroidism was diagnosed when free T4 was below < 0.6 ng/dL, along with low or inappropriately normal TSH. Central hypogonadism was defined by total testosterone < 250 ng/dL or estradiol < 20 pg/mL accompanied oligo- or amenorrhea, along with low or inappropriately normal serum LH and FSH.

### 2.1. Treatment Parameters

Radiosurgery was administered using a CyberKnife M6 platform, with the Multi-Plan system, to develop the planning treatments (Accuray Incorporated Sunnyvale, Sunnyvale, CA, USA) for all treatments in Mexico City, the Oncology Hospital at the National Medical Center. After a patient was accepted to be treated, the IMR and CT for planning were obtained. The median radiation dose was of 23.5 Gy (range 22–25 Gy), delivered in a single day, or a maximum of five days, in 5 Gy to 22 Gy per fraction. The individual Radiosurgery radiation protocol was decided by the radiation oncologist and neurosurgeon, based on the availability of appointments and the specific circumstances of each patient. For instance, in patients living out of town, the Institution provided accommodation for the duration of their treatments. Different organs at risk were carefully protected and all passed the Normal Tissue Constraints, according to the R.D. Timmerman charts. Medical treatments with SSA or DA were suspended at least one month before and during radiotherapy [19,20].

### 2.2. Hormonal Measurements

Assays for the measurement of GH and IGF-1 have changed throughout the follow-up of the cohort. Since 2007 to date, hormonal measurements were carried out using the same commercially available immunoassays. GH was measured by means of the Immulite, 2-site chemiluminescent assay (DiaSorin–Liaison, Saluggia, Italy), which has a detection limit of 0.009 ng/mL and an intra-and-interassay coefficient of variations of 2.5% and 5.8%, respectively. The International Reference Preparation (IRP) used in this GH assay was that of the World Health Organization (WHO), second 95/574. IGF-1 was measured by means of a 2-site chemiluminescent assay (DiaSorin–Liaison). The IRP in these IGF-1 assays was the WHO second 02/254. We established our own age-adjusted normative IGF-1 data, analyzing serum samples from 400 healthy adults, with an age range of 18 to 80 years, as previously described [4]. The hormonal assays used in prior years were: before 1999: RIA (LD 0.7) for GH and IRMA for IGF-1; 2000–2007: IMMULITE (LD 0.01) for GH and DIAGNOSTIC SYSTEM LAB for IGF-1.

### 2.3. Statistical Analysis

Descriptive and inferential statistics were used for data analysis, taking into account measures of central tendency and dispersion, according to the distribution of the variables. The Shapiro–Wilk test was used to determine the normality of the quantitative variables’ distribution. For the comparison of variables in independent groups, frequencies and proportions, Pearson’s Xi^2^ test, or Fisher’s exact test were used according to the expected value; and for quantitative variables, Student’s *t* test or Mann–Whitney U test were used, according to the type of distribution. For the comparison of variables between three dependent groups, Cochran’s Q test was used for qualitative variables, while for the comparison of quantitative variables, the Friedman’s test was used.

A comparative analysis of the baseline characteristics (before radiosurgery) between the groups of patients, with remission and without biochemical remission (at the last follow-up), was performed. Subsequently, through a crude and adjusted Cox Proportional Hazards Regression Analysis, we estimated the magnitude of association of the following variables before radiosurgery: age, gender, IGF-1 x ULN, GH, tumor size, and invasion of the cavernous sinus (grade 1–4 of Knosp’ classification), taking no biochemical remission of the disease at the last of follow-up as the outcome. A two-sided *p* value was used for the in-between group difference with respect to the primary outcome. A *p* value of *p* < 0.05 was considered statistically significant. The statistical software used was the Stata SE software version 16 (StataCorp, College Station, TX, USA).

## 3. Results

### 3.1. Baseline Characteristics

Of 265 patients with biochemical activity of acromegaly, 57 patients met the criteria for radiosurgery. Of the 57 patients analyzed, the mean age at diagnosis and at the time of radiosurgery was 47.1 ± 13.4 years and 54.5 ± 12.3 years, respectively, with a female predominance of 56.1%; 76.3% of patients had macroadenoma. The median follow-up from radiosurgery treatment was four years (IQR, 2–7.2 years). The delay in diagnosis of acromegaly in the studied cut-off was of five years (IQR, 4–8 years); all patients underwent transsphenoidal resection for tumor demassification and subsequently received radiosurgery as adjuvant treatment. The median delay time to radiosurgery was 38 months (IQR, 21–61 months). Median IGF-1 was 595.3 ng/mL ± 274.9 ng/mL and median GH was 5.66 ng/mL (IQR 2.5–18.3) before radiosurgery. The proportion of patients with tumoral invasion to the cavernous sinus was 78.95%. Pituitary hormone deficiencies and other patient characteristics are shown in Table 1. 

Of the patients referred for radiosurgery treatment, three patients (5.26%) were treated with the hypofractionated modality and 54 patients (94.74%) with a single dose; no statistically significant differences were observed in the biochemical remission rate after radiosurgery when comparing single dose vs. hypofractionated. Regarding the characteristics of radiosurgery treatment, of the total number of patients included in the study (57), it was only possible to obtain information on 37 patients; these results are presented in Table S1 of the Supplementary Materials.

### 3.2. Endocrine Outcomes

The biochemical remission rate was 45.6% (26 patients); of which 33.33% (19 patients) achieved biochemical control and 12.28% (seven patients) achieved biochemical cure. A progressive and statistically significant decrease was found in the comparison of IGF-1, IFG-1 x ULN, and basal GH concentrations at one year and at the end of follow-up. Likewise, statistically significant increases were observed in the percentage of patients who reached the control goals at the different evaluation times (Table 2). After radiosurgery, a significant reduction was observed in the percentage of patients using pharmacological therapy (Table 2). The LC tumor rate obtained in the group of patients evaluated was 100% (57 patients). 

Medical treatment after radiosurgery was characterized by an increase in the number of patients with no treatment at the end of follow-up (1.75% at baseline vs. 15.79 at the end of follow-up); 56.14% of patients were treated with somatostatin analogues (Octreotide LAR or Lanreotide autogel) before radiosurgery, a proportion that decreased to 49.12% at the end of follow-up. 36.84% of patients received treatment with somatostatin analogues (Octreotide LAR or Lanreotide autogel) plus cabergoline, a proportion that decreased to 28.07% at the end of follow-up. Finally, 5.26% received treatment with carbegoline alone, a proportion that increased to 7% at the end of follow-up.

### 3.3. Radiosurgery Safety Profile

Regarding hypothalamic-pituitary hormonal deficiencies after radiosurgery, it was observed that the percentage of hypocortisolism, hypothyroidism, and hypogonadism increased significantly throughout follow-up (Table 2). The reported rate of panhypopituitarism was 1.75% (1 patient) at baseline and 24.56% (14 patients) at the end of follow-up (*p* < 0.001). During follow-up after radiosurgery, two patients (3.5%) presented central nervous system tumors of the meningioma type. No cases of optic neuritis associated with radiation toxicity were observed. There were no cases of stroke.

A sub-analysis was carried out in which the 37 patients who had the radiosurgery parameters were included; in this analysis, the radiosurgery parameters were contrasted against the presence or absence of new endocrine deficiencies of the hypothalamic-pituitary axis at the end of follow-up, in which no statistically significant differences were observed. These results are presented in Table S2 of the Supplementary Materials.

### 3.4. Bivariate Analysis between Active and Biochemical Control Groups

In the bivariate analysis between the groups of patients without and with biochemical remission of acromegaly, no significant differences were observed between the baseline characteristics of the patients (Table 3).

### 3.5. Multivariate Analysis

In a Cox Proportional Hazards Regression Analysis, only the baseline IGF-1 x ULN concentration above range was shown to be a risk factor (HR of 1.33; 95% CI 1.01–1.88) for no biochemical remission. Invasion of the cavernous sinus by the growth hormone-producing tumor (grade 1–4 of Knosp’ classification) had a 2.53 (95% CI 0.92–6.97) for no biochemical remission. The rest of the variables had no statistically significant association with this lack of biochemical control (see Table 4).

## 4. Discussion

Currently, the treatment of acromegaly contemplates a multimodal approach, which initially includes surgical and medical treatment (with SSA and DA) [13]. However, despite the existence of these therapeutic tools, high persistence rates of biochemical activity have been reported, so that in such scenarios the use of high-cost drugs, such as Pasireotide and Pegvisomant, are suggested [21,22]. Unfortunately, in low-income countries, these resources are not available in public health institutions, which has led to the use of third-line therapeutic alternatives such as fractionated stereotactic radiotherapy and radiosurgery, whose results have been variable in relation to local tumor control, safety profile, and methodology in the measurement of outcomes [23]. Therefore, the aim of this study was to establish the efficiency and safety of radiosurgery (CyberKnife) as an adjuvant treatment in the multimodal approach of patients diagnosed with acromegaly, as well as to determine which factors were associated with the biochemical persistence of the disease. 

In the cohort evaluated, a biochemical remission rate of 12.2% was observed, 33.3% of patients were in biochemical control with medical treatment, and 54.4% persisted with biochemical activity; these data are similar to those reported by Iwata H. et al., with endocrine remission in 17.3% (95% CI 7.02–27.58) of their cases, according to the curtain criteria [24]. Similarly, Ehret et al. found a biochemical remission rate in 18% of their patients, biochemical control with medical treatment in 48%, and 34% remaining active [25]. In our study, we obtained percentage reduction rates of both total IGF-1 and IGF-1 x ULN of 70.15% and 65.5%, respectively, similar to those found by Ehret et al., who observed a reduction of total IGF-1 and IGF-1 x ULN at the end of their follow-up of 48.5% and 48.5%, respectively [25]. Our LC rate was 100% at the last visit, equal to that reported by Ehret et al. [25] and similar to Iwata with 82.5% (95% CI 72.17–92.83%) [26]. 

Roberts, BK. et al. analyzed a retrospective series of nine patients where they found that 44.4% achieved biochemical remission, which was defined as a normalization of IGF-1 concentrations, at the end of follow-up, and 44.4% had persistence of biochemical activity; the mean follow-up was 17.8 months [27]. Sala E. et al. conducted a study of 22 patients where an IGF-1 normalization rate of 31.5% was reported after CyberKnife radiosurgery treatment at six months follow-up, while 54.5% of patients remained with active disease; the study remission rate at 50 months follow-up was 50% [28]. Such results cited above are consistent with the effectiveness reported in our study, where 33.3% achieved an IGF-1 < 1.2 × ULN and 45.6% achieved endocrine remission at the last visit (GH < 1 ng/mL AND IGF-1 < 1.2 × ULN). Singh, R. et al. performed a meta-analysis involving a total of 1533 patients with acromegaly treated with radiosurgery in the modalities (LINAC, CyberKnife, and GammaKnife), in which they found endocrine remission and endocrine control rates of 43.2% (95% CI 31.7–54.6%) and 55% (95% CI 27.6–82.4%), respectively, at five years of follow-up. The estimated local control rate at 10 years was 92.8% (95% CI 83–100%) [23]. 

In relation to the safety of radiosurgery found in our study at the end of follow-up, an increase in the rates of hypothyroidism of 28% and hypocortisolism of 26% was obtained; panhypopituitarism presented an increase of 22.8%. These data differ from those obtained by Eheret et al., who found an increase in the rates of hormone deficiencies in relation to hypogonadism of 4%, of hypocortisolism of 4%, and of hypothyroidism of 6% after radiosurgery at the end of follow-up, rates of hypothalamic-pituitary deficiencies [25]. Salas et al. reported an increased rate of hypothalamic-pituitary hormone deficiencies of 22.8% at the end of follow-up (5 years) that affected at least one hormonal axis [28]. The above were lower than those found in our study. The results of the meta-analysis by Singh R. et al., which included different radiosurgery modalities for the treatment of acromegaly, estimated an overall rate of new hypothalamo–hypophysiary deficiencies of 26.8% (95% CI 16.8–36.7%) [23]. In relation to hypocortisolism, in our clinical practice, we used a cut-off point of <5 mcg/dL accompanied by symptoms and signs of adrenal insufficiency. Patients in whom there was doubt about the diagnosis were referred for induced hypoglycemia testing. However, most patients had cortisol levels between 3 and 15 mcg/dL and the diagnosis can only be made with stimulation tests such as the Synacthen test, which we did not have in our center. Therefore, it is likely that the prevalence of hypocortisolism in our population has been underestimated.

In relation to optical radiation toxicity and cerebral vascular events, our results are in agreement with other studies such as that of Roberts B. et al. [27] and Iwata H. et al., [26] who reported absences of this complication in their cohorts. However, the rates of radiosurgery-associated visual toxicity reported in its different modalities (LINAC, Gamma, CyberKnife) are variable, ranging from 0% to 5%, with a pooled rate 2.7% (95% CI 1.3–4.2%) [23].

Regarding potential predictors for the absence of biochemical remission following radiosurgery, we found a higher probability of no biochemical remission when the baseline IGF1-1 x ULN value was elevated, with an adjusted HR of 1.33 (95% CI 1.01–1.88). Similarly, Ehret F. et al. found that elevated pretreatment IGF-1 x ULN values were associated with a lower likelihood of biochemical remission of acromegaly [25]. These data are also congruent with those obtained in several studies that evaluated the association between elevated IGF-1 x ULN and total IGF-1 levels before surgical treatment and/or fractionated stereotactic radiotherapy, finding that elevated baseline concentrations were predictive of a lack of biochemical remission [25,29,30,31]. On the other hand, the presence of tumoral invasion into the cavernous sinus (grade 1–4 of Knosp’ classification) showed a tendency to statistical significance with HR of 2.53 (0.92–6.97) as a risk factor for no biochemical remission, a finding previously reported in invasive adenomas that have a low probability of cure and/or remission [30]. All of the above is a consequence of the difficulty in the surgical dissection of somatotroph cells in the cavernous sinus. 

CyberKnife is a relatively new technology for frameless stereotactic radiosurgery, in which a mobile linear accelerator is mounted on a robotic arm with an image-guided robotic system. Patients are immobilized in a thermoplastic mask and radiation doses can be delivered in single or multiple fractions with a target accuracy of 0.5 to 1 mm, similar to that achieved with frame-based stereotactic radiosurgery [32]. Radiosurgery in its CyberKnife modality is an adjuvant strategy to surgery and a medical treatment in acromegaly with an acceptable effectiveness profile where, according to the series studied, biochemical control ranges from 17–65.4%, with optimal tumor control rates corresponding to ranges of 96–100% [32]. Radiosurgery shows a variable safety profile, in relation to hypothalamic-pituitary deficiencies ranging from 7.8–57% of hypopituitarism and visual deficit rates from 0–11.1% [32]; the variability in its safety makes it necessary to carry out studies to establish it as a treatment tool that can be widely used in the management of the disease. 

The weaknesses of our study are firstly related to the sample size, although the study population is representative of the patients under follow-up in our clinic; there was also lack of information regarding treatment parameters with radiosurgery, which would be important for the evaluation of new hormonal deficits, especially the dose administered to the pituitary stalk; and the median follow-up after radiotherapy was short, so that the efficacy and side effects could be underestimated, indicating that long-term studies are required to evaluate the efficacy and safety outcomes of CyberKnife radiosurgery. Its strengths are that data are presented from a cohort of a well-characterized rare disease, with a pre-established diagnostic and therapeutic protocol from the beginning of the acromegaly clinic, reducing the probability of some biases.

## 5. Conclusions

The results of our study suggest that radiosurgery (CyberKnife modality) is a safe and effective technique in the adjuvant treatment of GH-producing tumors. Additionally, elevated pre-radiosurgery IGF x ULN levels could be a predictor of a lack of biochemical remission of acromegaly.

## Figures and Tables

**Table 1:** Baseline characteristics of patients with acromegaly treated with radiosurgery (*n* = 57).

Variable	*n* = 57
Female gender, No. (%)	32 (56.14)
Age, mean ± SD, yrs	47.1 ± 13.4
Macroadenoma, No. (%)	42 (76.36)
Age at which it was radiated, mean ± SD, yrs	54.5 ± 12.3
Delay in diagnosis, median (IQR), yrs	5 (4–8)
Time from surgical treatment to radiotherapy, median (IQR), moths	38 (21–61)
Baseline IGF 1, mean ± SD, ng/mL	595.32 ± 274.94
IGF 1 X ULN, mean ± SD	2.96 ± 1.37
GH, median (IQR), ng/mL	5.66 (2.5–18.3)
Cavernous sinus invasion, No. (%)	45 (78.95)
Medical treatment of acromegaly	56 (98.25)
Hormone deficiencies, No. (%)	
Hypothyroidism	15 (26.32)
Hypocortisolism	8 (14.04)
Hypogonadism	12 (21.05)
Comorbidities, no. (%) *	
CNS tumor	1 (1.75)
Optic neuritis	1 (1.75)
Stroke	2 (3.51)
T2D	19 (33.33)
Carbohydrate intolerance	19 (33.33)
Hypertension	22 (38.6)
Dyslipidemia	6 (10.53)
Heart disease	3 (5.26)

Abbreviations: SD, standard deviation; IQR, interquartile range; yrs, years; CNS, central nervous system; T2D, type 2 diabetes. * The comorbidities shown were present before radiosurgery.

**Table 2:** Analysis of the evolution of hormonal parameters and deficiencies of the hypothalamus-pituitary axis at the baseline, at year and at the end of follow-up (*n* = 57).

Variable	Baseline (*n* = 57)	1 Year (*n* = 57)	Last Follow Up (*n* = 57)	*p*
IGF-1, median (IQR), ng/mL	605.87 (410.8–761.4)	285.9 (208.9–416.4)	181 (117.2–270)	<0.001 ^a^
IGF-1 x ULN, median (IQR)	2.87 (1.98–3.85)	1.39 (1.1–2.2)	0.99 (0.72–1.3)	<0.001 ^a^
GH, median (IQR), ng/mL	5.66 (2.5–18.3)	1.69 (1.16–2.6)	0.77 (0.47–1.59)	0.001 ^a^
% reaching bGH < 2.5 ng/mL, No. (%)	14 (24.56)	41 (71.93)	9 (10.23)	<0.001 ^b^
% reaching bGH < 1 ng/mL, No. (%)	8 (14.04)	12 (21.05)	53 (60.23)	<0.001 ^b^
% reaching IGF-1 < 1.2 × ULN, No. (%)	7 (12.28)	19 (33.33)	40 (70.18)	<0.001 ^b^
% reaching bGH < 1 ng/mL AND IGF-1 < 1.2 × ULN, No. (%)	4 (7.02)	6 (10.53)	26 (45.61)	<0.001 ^b^
% requiring pharmacological therapy, No. (%)	56 (98.25)	53 (98.15)	48 (84.21)	<0.001 ^b^
Hormone deficiencies, No. (%)				
Hypothyroidism	15 (26.32)	20 (35.08)	31 (54.34)	<0.001 ^b^
Hypocortisolism	8 (14.04)	10 (17.54)	23 (40.35)	<0.001 ^b^
Hypogonadism	12 (21.05)	14 (24.56)	27 (47.37)	<0.001 ^b^

Abbreviations: IQR, interquartile range. ^a^ *p* value estimated with Friedman’s test, ^b^ *p* value estimated with Cochran’s Q test.

**Table 3:** Comparison of baseline characteristics between the active and non-active groups at the end of follow-up (*n* = 57).

Variable	Without Biochemical Remission (*n* = 31)	Biochemical Remission (*n* = 26)	*p*
Female gender, No. (%)	15 (48.39)	17 (65.38)	0.19 ^a^
Age, mean ± SD, yrs	52.45 ± 13.39	57 ± 10.61	0.16 ^b^
Time from surgical treatment to radiotherapy, median (IQR), moths	5.56 (2.6–22.6)	4.85 (2.1–15)	0.18 ^d^
Baseline IGF 1, mean ± SD, ng/mL	639.55 ± 292.01	542.59 ± 248.36	0.18 ^b^
Time from radiosurgery until last evaluation, median (IQR), years	3.16 (2.09–6.5)	5.9 (2.39–7.17)	0.10 ^d^
IGF 1 X ULN, mean ± SD	3.12 ± 1.41	2.78 ± 1.33	0.36 ^b^
GH, median (IQR), ng/mL	5.66 (2.6–22.6)	4.85 (2.1–15)	0.18 ^d^
Cavernous sinus invasion, No. (%)			
Knosp grade 1 to 4	24 (77.42)	21 (80.77)	0.75 ^a^
Knosp grade 3 and 4	16 (51.61)	10 (38.46)	0.32 ^a^
Hormone deficiencies, No. (%)			
Hypothyroidism	7 (22.58)	8 (30.77)	0.48 ^a^
Hypocortisolism	2 (6.45)	6 (23.08)	0.12 ^c^
Hypogonadism	7 (22.58)	5 (19.23)	0.75 ^a^

Abbreviations: Abbreviations: SD, standard deviation; IQR, interquartile range; yrs, years. ^a^ *p* value estimated with Pearson’s Xi^2^, ^b^ *p* value estimated with Student’s *t* test, ^c^ *p* value estimated with Fisher’s exact test, ^d^ *p* value estimated with Mann-Whitney U test.

**Table 4:** Cox Proportional Hazards Regression Analysis for the absence of biochemical remission of the disease after radiosurgery (*n* = 57).

Variable	Crude HR	95% CI	*p*	Adjusted HR **	95% CI	*p*
Female gender	0.53	0.25–1.11	0.09	0.55	0.26–1.18	0.12
Age *	0.98	0.95–1.00	0.17	0.97	0.93–1.00	0.07
Baseline IGF-1 x ULN *	1.41	1.03–1.94	0.03	1.33	1.01–1.88	0.04
Baseline GH *	0.99	0.99–1.00	0.62	0.99	0.98–1.01	0.81
Tumor volume prior to radiosurgery, mm^3^ *	0.99	0.99–1.00	0.73	0.99	0.99–1.00	0.56
Knosp grade 1 to 4	2.24	0.89–5.62	0.083	2.53	0.92–6.97	0.07
Knosp grade 3 to 4	1.16	0.56–2.42	0.67			

Abbreviations: HR, hazard ratio. * Included as continuous variable. ** Cox proportional hazard model including female gender, age, baseline IGF-1 X ULN, baseline GH, tumoral volume and Knops 1 to 4 grade.

## Data Availability

The datasets used and/or analyzed during the current study are available from the corresponding author on reasonable request.

## Supplementary Materials

The following supporting information can be downloaded at: https://www.mdpi.com/article/10.3390/cancers15051438/s1, Table S1: Radiosurgery treatment characteristics. Table S2: Comparison of radiosurgery treatment characteristics between the with and without hormone deficiency groups at the end of follow-up.
