# Supplementary material for: Efficiency and Safety of CyberKnife Robotic Radiosurgery in the Multimodal Management of Patients with Acromegaly
Source: Cancers (Basel). 2023 Feb 24;15(5):1438. doi: 10.3390/cancers15051438 (PMC10001340; doi:10.3390/cancers15051438)
Supplement: Supplementary file 1 [file cancers-15-01438-s001.zip › cancers-2228111-supplementary.pdf]

Supplementary Materials

Table S1.- Radiosurgery treatment characteristics.

Variable	Mean	Median	IQR	SD
Prescribed dose, cGy	2302.94	2300	2200-2500	266.83
Maximum dose, cGy	3002.09	2942.18	2682.92-3333.33	480.96
Coverage %, %	96.88	96.41	95.77-98.25	1.39
Isodose curve, %	77.5	77	72-84	7.52
GTV, cc	1.28	0.83	0.47-1.52	1.19
Chiasm dose, cGy	966.45	939.48	873.26-1059.78	275.37
Right optic nerve dose, cGy	636.62	620.46	346.16-908.35	319.67
Left optic nerve dose, cGy	706.8	735.14	497.1-944.79	292.53
Brain dose, cGy	1913.15	1555.16	912.17-3048.17	1158.93

Abbreviations: cGy, centigray; SD, standard deviation; IQR, interquartile range.

Table S2.- Comparison of radiosurgery treatment characteristics between the with and without hormone deficiency groups at the end of follow-up (n=37).

Variable, median (IQR)	With hormone deficiency* (n=12)	Without hormone deficiency* (n=25)	P[a]
Prescribed dose, cGy	2300 (2250-2450)	2300 (2300-2500)	0.49
Maximum dose, cGy	2857 (2711-3112)	2973 (2817-3472)	0.26
Coverage %, %	97.5 (96.3-98.7)	96 (95.7-98)	0.05
Isodose curve, %	80 (76-85)	77 (72-83)	0.13
GTV, cc	0.9 (0.71-1.4)	0.8 (0.45-1.49)	0.41
Chiasm dose, cGy	912 (863-983)	950 (876-1060)	0.2
Right optic nerve dose, cGy	790 (366-982)	618 (450-840)	0.32
Left optic nerve dose, cGy	635 (474-800)	737 (676-965)	0.19
Brain dose, cGy	1512 (1325-2333)	1555 (912-2597)	0.76

Abbreviations: cGy, centigray; SD, standard deviation; IQR, interquartile range. [a]P value estimated with Mann-Whitney U test. *Hormone deficiency was defined as the presence of at least one new hormonal deficiency of the hypothalamic-pituitary axis.
